# Breaking barriers in the career development of women in academic psychiatry

**DOI:** 10.1192/bjo.2024.808

**Published:** 2024-11-12

**Authors:** Mariana Pinto da Costa, Silvana Galderisi, Helen Herrman, Anita Riecher-Rössler, Danuta Wasserman

**Affiliations:** Institute of Psychiatry, Psychology & Neuroscience, King's College London, UK; and Institute of Biomedical Sciences Abel Salazar, University of Porto, Portugal; Department of Mental and Physical Health and Preventive Medicine, University of Campania Luigi Vanvitelli, Italy; Orygen and the Centre for Youth Mental Health, The University of Melbourne, Australia; Medical Faculty, University of Basel, Switzerland; Karolinska Institute, Stockholm, Sweden

**Keywords:** Barriers, professional development, academic psychiatry, women, gender

## Abstract

Academic psychiatry is essential for advancing mental health understanding and treatments. However, women encounter more obstacles hindering their progress in academia than men. This Editorial aims to highlight these obstacles and propose strategies to address them, advocating for a more supportive environment for women psychiatrists’ ongoing growth and development. The importance of supportive environments, fair access to opportunities and structural changes, including initiatives for mentorship, funding and flexible work arrangements, are crucial. Collaboration among governments, institutions and organisations is needed to enhance research infrastructure and promote gender equality. Encouraging and recognising women's contributions in research fosters inclusivity and innovation. Prioritising these efforts is vital for the existence, well-being and success of women in academic psychiatry.

Academic psychiatry plays a crucial role in advancing our understanding of mental health conditions, developing innovative treatments and training the next generation of professionals. Professional development is essential for ensuring high-quality care and staying abreast of advancements in research and practice. However, many psychiatrists work in settings with limited resources, hindering their ability to access continuing education opportunities, attend conferences and engage in research.^1^ Psychiatrists also face high rates of burnout and turnover because of the demanding nature of the work, heavy case-loads and the emotional toll of patient care.^2^ Promoting work–life balance, providing access to mental health support services and fostering a supportive work environment are vital for retaining psychiatrists and facilitating their professional growth. Streamlining complex regulatory requirements and administrative burdens can free up psychiatrists’ time and energy, allowing them to focus more on professional development activities such as research, education and other collaborations. Still, psychiatrists of all genders have limited access to (high-quality) professional development. Such access is crucial for staying up to date on the latest research findings, treatment modalities and best practices, particularly for those in remote or underserved areas. Mentorship plays a pivotal role in the professional development of academic psychiatrists, and can provide both men and women with valuable guidance, support and opportunities for career advancement.

The European Commission's Directorate-General for Research and Innovation recently reported that in every field of research and development, women represented no more than a third of the highest-ranking staff.^3^ In academia, women encounter more obstacles and gender-related barriers that hinder their career progress. Women academic physicians face slower promotion rates and inconsistent or non-comprehensive paid family leave policies.^4^ Women medical researchers are less likely than men to receive independent funding or publish in high-impact journals.^5^ Recent data from the Association of American Medical Colleges shows that women comprise 59% of faculty at the instructor level, 47% at the assistant professor level, 38% at the associate professor level and 25% at the full professor level.^6^ Women are also less likely to receive professional titles and prestigious awards, and are not well-represented among journal boards and medical society leadership roles.^7^ Women researchers are affected by gender inequities in funding, career development awards and start-up packages. In psychiatry, women do worse than comparably qualified men in salary, promotions, grants and scholarly publishing.^8^ Women academic psychiatrists may face unfair treatment in their career progression.^9^ For instance, they might be denied flexible working arrangements to reduce their clinical working time when their male colleagues have similar requests approved; they might not be authorised to integrate additional institutional leadership responsibilities into their job plans; and, when on maternity leave, they may have their research funding applications appropriated by their colleagues without their involvement or permission.

Authorship plays a critical role in academic psychiatry, greatly influencing career advancement, recognition and promotion within academia. First and last authorship positions are particularly valued, as they often reflect leadership in the research process. However, women in academic psychiatry face challenges in securing authorship opportunities compared with men. Research shows that women are underrepresented as first and last authors in high-impact journals and are less likely to be credited as lead researchers, even when they make significant contributions to collaborative projects. Although there has been some progress toward near parity in first authorship for women in psychiatry journals, their transition to senior authorship is slower and they remain underrepresented as senior authors.^10^ This disparity can reduce women's visibility in academia, limit their eligibility for prestigious awards and hinder their career progression. Ensuring equitable access to authorship opportunities is essential for achieving gender parity in academic psychiatry. Institutions should implement clear guidelines for authorship contributions, promote transparency in the allocation of research credit and encourage mentorship schemes that support women in attaining senior authorship roles.

More women than men lack access to formal mentorship programmes, role models or experienced mentors within institutions, professional organisations and academic settings.^11^ Previous research has shown that single women psychiatrists were the most likely of all to choose academic reasons as their top reason to leave the country, but when women were in a relationship or had children, their attitude toward migration changed.^12^ However, for men, the top reason to leave the country was finances, and this did not change depending on their relationship or parenthood status.

A recent survey of psychiatrists in Europe reported specific external and internal barriers faced by women. Women reported higher rates of gender discrimination in their professional development and a lack of institutional commitment to diversity and inclusion initiatives.^13^ They described negative attitudes to self-promotion and networking, and believing they lack the skills to promote their accomplishments.^13^ Therefore, effective strategies must encompass both individual and structural dimensions. It is essential to enhance women's self-confidence and perceptions of their abilities. This requires training to increase their skills in self-promotion and networking. Simultaneously, structural reforms within workplace environments are indispensable in mitigating gender-based inequities. These include rectifying discrepancies in promotion rates, salary scales, professional opportunities and task assignments between genders. In 2024, some panels at international psychiatry congresses are still composed exclusively of men, a phenomenon referred to as ‘manels’. This highlights how far away we still are from achieving gender parity in academic psychiatry.

Resource allocation is another critical consideration, with staff salaries usually representing a significant proportion of the budgetary expenditure in services. Ensuring gender equity in fellowships, training opportunities and career positions is of utmost importance. Women's participation in research has historically been hindered by systemic barriers, including unequal access to funding and career advancement opportunities.^7^ Therefore, prioritising the availability of fellowships, training and career positions for women is essential not only for promoting gender diversity, but also for fostering a more inclusive and equitable research environment. By addressing disparities in funding and career advancement, research institutions can harness the full potential of their workforce, leading to greater innovation and productivity. Investing in gender equity initiatives within research programmes is not only a matter of social responsibility, but also a strategic imperative for driving progress and excellence in scientific endeavours.

Building a more equitable future for research requires ensuring fair and equal access to opportunities for women. However, women academic psychiatrists often face stigma, prejudice and misconceptions about their work, which can hinder their professional advancement. Women junior faculty reported greater susceptibility to stereotype threat, sensitivity to rejection and identification with their gender (or group identification more generally), as well as feelings of lower relative potential and a reduced sense of belonging compared with their male counterparts.^14^ Gender-related microaggressions primarily affect women in academia through three social mechanisms: gender blindness, gender-stereotypical assumptions and sexual objectification.^15^

Women play a crucial role in shaping research agendas, bringing to the table a unique perspective that enriches the breadth and depth of scholarly inquiry. Women's multifaceted viewpoints, shaped by life experiences, contribute to a more comprehensive understanding of research topics. Importantly, women's involvement in research has been recognised for its ability to foster inclusivity and equity within academic settings. A nuanced approach to problem-solving and decision-making can uncover insights that might otherwise remain hidden, thus broadening the scope and impact of research outcomes. Diverse research teams that include women tend to generate more innovative ideas and produce higher-quality research outputs.^16^ Integrating women's perspectives into research practices not only enhances the rigor and relevance of scholarly work, but also promotes a more holistic and inclusive research culture. As we continue to champion diversity and inclusivity in academia, recognising and valuing the unique contributions of women in research is essential for advancing knowledge and addressing the complex challenges facing society today. Women's involvement in research is essential not only for fostering inclusivity and equity within academic settings, but also for ensuring the full utilisation of the world's talent pool. Leaving women out is a matter of injustice and a detriment to the advancement of knowledge. Challenges such as institutional barriers related to mentoring, time management, the impact of bias, exclusion from formal and informal networks, and involvement in committees and other activities must be addressed.

Addressing environmental barriers, such as the lack of opportunities and support, and combating gender discrimination in professional settings, are imperative steps toward fostering inclusive workplaces. Women often face higher workloads not only from professional demands, but also because of traditional gender roles that assign disproportionate responsibilities in caregiving and household management. This double or triple burden contributes to burnout and turnover, exacerbated by administrative burdens and higher pastoral expectations, often placed on women. Removing these barriers and ensuring a foundation of empowerment and equity early in women's education and professional development is paramount. High-income countries possess the resources to spearhead and champion initiatives that promote gender equality and foster inclusive work environments in academic psychiatry.

Overcoming the obstacles that women face for their professional development in academic psychiatry requires a concerted effort from institutions, professional organisations and individual practitioners. Initiatives like the Athena Scientific Women's Academic Network (SWAN) exemplify good practices in promoting gender equality by fostering an inclusive culture and providing a framework for institutional improvements. The literature often considers women as a homogenous group, overlooking the unique experiences of women with different characteristics – for example, with respect to their ethnicity, pregnancy, maternity, sexual orientation or gender identification – and does not account for nuanced differences between groups. For example, although all women are susceptible to multiple forms of harassment, the experiences of women who are Black or minority ethnic and LGBTQ+ may be distinct, facing racialised and sexist stereotypes, and being perceived as either overly aggressive or angry or insufficiently assertive, which can further silence them.^9^ This lack of differentiation can lead to generalised conclusions that fail to address the specific needs and challenges faced by these different groups.

By tackling stigma, allocating resources, preventing burnout, streamlining administrative processes, improving access to continuing education and enhancing mentorship opportunities, we can create a more supportive environment for the recruitment and career development of women in academic psychiatry. It is imperative that we prioritise these efforts to ensure the well-being of professionals in academic psychiatry, independent of gender, which will positively impact patients’ clinical care.

## Data Availability

Data availability is not applicable to this article as no new data were created or analysed in this Editorial.
